# Research hotspots and frontiers in agricultural multispectral technology: Bibliometrics and scientometrics analysis of the Web of Science

**DOI:** 10.3389/fpls.2022.955340

**Published:** 2022-08-11

**Authors:** Yali Zhang, Dehua Zhao, Hanchao Liu, Xinrong Huang, Jizhong Deng, Ruichang Jia, Xiaoping He, Muhammad Naveed Tahir, Yubin Lan

**Affiliations:** ^1^College of Engineering, South China Agricultural University, Guangzhou, China; ^2^National Center for International Collaboration Research on Precision Agricultural Aviation Pesticide Spraying Technology, Guangzhou, China; ^3^Department of Information Consulting, Library, South China Agricultural University, Guangzhou, China; ^4^Department of Agronomy, Pir Mehr Ali Shah-Arid Agriculture University, Rawalpindi, Pakistan; ^5^College of Electronic Engineering and College of Artificial Intelligence, South China Agricultural University, Guangzhou, China

**Keywords:** multispectral, agriculture, CiteSpace, remote sensing, Web of Science, NDVI

## Abstract

Multispectral technology has a wide range of applications in agriculture. By obtaining spectral information during crop production, key information such as growth, pests and diseases, fertilizer and pesticide application can be determined quickly, accurately and efficiently. The scientific analysis based on Web of Science aims to understand the research hotspots and areas of interest in the field of agricultural multispectral technology. The publications related to agricultural multispectral research in agriculture between 2002 and 2021 were selected as the research objects. The softwares of CiteSpace, VOSviewer, and Microsoft Excel were used to provide a comprehensive review of agricultural multispectral research in terms of research areas, institutions, influential journals, and core authors. Results of the analysis show that the number of publications increased each year, with the largest increase in 2019. Remote sensing, imaging technology, environmental science, and ecology are the most popular research directions. The journal *Remote Sensing* is one of the most popular publishers, showing a high publishing potential in multispectral research in agriculture. The institution with the most research literature and citations is the USDA. In terms of the number of papers, Mtanga is the author with the most published articles in recent years. Through keyword co-citation analysis, it is determined that the main research areas of this topic focus on remote sensing, crop classification, plant phenotypes and other research areas. The literature co-citation analysis indicates that the main research directions concentrate in vegetation index, satellite remote sensing applications and machine learning modeling. There is still a lot of room for development of multi-spectrum technology. Further development can be carried out in the areas of multi-device synergy, spectral fusion, airborne equipment improvement, and real-time image processing technology, which will cooperate with each other to further play the role of multi-spectrum in agriculture and promote the development of agriculture.

## Introduction

Precision agriculture is currently in a phase of rapid development, which integrates technologies such as remote sensing, big data, and decision analysis, and aims to achieve efficient use of resources, rational inputs, and co-benefits in environmental and economic terms through variable and controllable scale farm management (Mazzia et al., 2020). Whereas information acquisition is the basis of precision agriculture, farming information is precisely the dynamic tracking of the agricultural environment and the state of plants at various growth periods (Huang et al., 2016), and plant phenotypes are important expressions of information on morphological characteristics that can be observed from plants (Huang et al., 2020). Both are important expressions of information in precision agriculture. Therefore, farming information and plant phenotypes reflect the information status of plants and provide reliable information for decision makers. In this case, how to obtain the required information becomes critical.

The advent of multispectral technology provides an effective and fast way to obtain agricultural information or plant phenotype information. Spectral imaging technology (Garini et al., 2006) emerged in the 1960s and was early applied in remote sensing, military and other fields. With the emergence of semiconductor photodetectors, spectral technology developed rapidly and its applications were extended to agriculture, environmental science, food engineering and other fields. Multispectral imaging technology is a kind of fusion technology of image and spectrum, which can acquire both spatial and spectral information of the object. In recent years, with the development of multispectral technology, its application in the field of agriculture has become more and more extensive. In the field of agriculture multispectral has been deeply applied in several aspects such as grain yield prediction (Zhou et al., 2017), pest and disease detection (Sankaran et al., 2010), nondestructiveness detection (Yu et al., 2018), remote sensing of agricultural drones Berni et al. (2009), weed identification (Pena et al., 2013; Sa et al., 2018), water content detection (Baluja et al., 2012), biomass (Kross et al., 2015), vegetation detection (Candiago et al., 2015), and inorganic matter detection (He et al., 2016). Agricultural multispectral technology is based on multidisciplinary fusion research, which makes use of data fusion techniques with multiple platforms, sensors, and remote sensing to provide data for research in the field of agriculture.

Bibliometrics and scientometrics are quantitative tools commonly used in scientific research. They are applied to analyze the frontiers of a topic or research field from macro- to micro-perspectives, which includes elements such as countries, institutions, authors, keywords, and journals (Raparelli and Bajocco, 2019; Zhang et al., 2019). These tools integrate computer engineering, big data applications, and statistics, and are widely applied in many fields (Chen, 2017) to provide rich assessments and analyses in different areas. The advantages of bibliometrics are reflected in stronger analytical efficiency for keyword analysis, research hotspot frontiers, and reference co-occurrence analysis. Scientometrics is an effective method for discovering research hotspots, and a powerful helper for researchers to understand the evolutionary path of research as well (Xie et al., 2020). It provides a systematic and comprehensive judgment.

The development and application of multispectral technology in agriculture promotes the development of precision agriculture and helps solve problems encountered in today’s agricultural development. The bibliometrics and scientometrics analysis was conducted in this paper by reviewing publications related to agricultural multispectral research in agriculture between 2002 and 2021 from the Web of Science (WOS). Multiple softwares, CiteSpace, VOSviewer, and Microsoft Excel were adopted for analyzing and mapping of scientific knowledge to characterize the research hotspots and frontiers of agricultural multispectral technology. Comprehensive analysis was discussed in terms of research areas, institutions, influential journals, core authors, and keywords. Multiple research areas on agricultural multispectral research were identified through keyword co-citation analysis. The main research directions were proposed through literature co-citation analysis as well.

## Materials and methods

### Literature search strategy

The WOS database was taken as the data source with multispectral as the theme. Agriculture multispectral research mostly focus on crops, soil, moisture, biomass, etc. Therefore, the search formula was determined as: TS = (multispectral)AND TS = (Agricultural UAV or agriculture or crop or tomato or corn or wheat or rice or citrus or cotton or soybean or moisture or soil or pest or weed or yield or potato or precision agriculture or Sugar cane or Nitrogen or tea or biomass or water fractions or Vegetables or Agricultural Remote Sensing or Chlorophyll or Pesticides). The literature data were searched for the time period from January 2002 to December 2021. Finally, 3,830 publication records were exported with each record containing author, title, source document, abstract, and cited references.

### Methodology

Data mining, analysis and visualization were conducted for 3,830 literature related to agricultural multispectral research through CiteSpace 5.8.3, VOSviewer, and Microsoft Excel.

CiteSpace software^1^ was developed by Prof. Chaomei Chen, Professor (tenure-track faculty) at School of Information Science and Technology, Drexel University, United States. The software is citation visualization and analysis software gradually developed in the context of scientometrics and data visualization (Chen, 2006; Chen et al., 2010). Thomas’ and Kuhn’s structure of scientific revolution provides the philosophical basis for CiteSpace. Another design inspiration for this software is a theory called structural holes, which was proposed by Burt at the University of Chicago in his study of social networks and social values (Chen, 2013). The software features dynamic complex network analysis and data visualization, and the visualization of CiteSpace can be divided into two main modes: cluster view and temporal view. The most prominent feature in CiteSpace is the co-citation analysis of the literature as a way to explore the knowledge structure of research. CiteSpace helps summarize clusters research frontiers and reveal the valuable knowledge points in the frontiers of agricultural multispectral research.

VOSviewer,^2^ afree JAVA-based software developed by VanEck and Waltman at the Centre for Science and Technology Studies (CWTS), Leiden University, the Netherlands, in 2009 (van Eck and Waltman, 2010), is mainly oriented toward documentary data, relational knowledge units of documents construction. It is adapted to the analysis of one-mode undirected networks and focus on the visualization of scientific knowledge. VOSviewer draws scientific knowledge maps to show the inter-relationships between literatures in agricultural multispectral research. The most valuable advantage of VOSviewer over other bibliometric software is its graphical presentation capabilities, its suitability for large-scale data, and the versatility in adapting to source data in various formats from various databases. VOSviewer also provides text mining capabilities for constructing and visualizing co-occurrence of important terms extracted from scientific literatures about agricultural multispectral research. VOSviewer also provides text mining capabilities for building and visualizing co-occurrence networks of important terms extracted from these literatures.

Keywords are the core summary of a scientific paper. Analysis of the keywords gives a glimpse of the topic of the paper as that keywords given in a paper must have some kind of association. This association can be expressed by the frequency of co-occurrence. It is generally believed that the more frequent a word pair appears in the same literature, the closer the relationship between the two themes. Co-occurrence analysis investigates the common occurrence of lexical pairs of nouns or phrases in a literature set to determine the relationship between themes in the disciplines represented by that literature set. By counting the frequency of occurrences of two theme terms in the same document, a co-word network of these word-pair associations can be formed. The analysis of keywords can explore the research themes and hotspots of the literatures. The statistics of the frequency of keywords can analyze the hotspots of the research field. Two or more papers are cited by one or more papers at the same time, then these papers will constitute a co-citation relationship, and the co-citation relationship of the literature will change with time. A research hotspot is the focus and concentration of a technical field over a period of time, which is manifested by the emergence of a large number of papers and patents on a technical issue. The concept of research hotspot was first introduced by Plath in 1965, and has been developed and extended over the past 60 years to multiple levels. Analyses of research hotspots help clarify the development history, correctly understand the research lineage, and provide reference for future directions of agricultural multispectral technology.

## Results

### Basic data information

The searched literatures were first processed and removed irrelevant ones, and a total of 3,830 publications and 53,390 references were obtained. The average of 191.5 publications per year from 2002 to 2021 was available in the field of agricultural multispectral research. From the analysis results it is clear that multispectral study is flourishing in agriculture. These literatures involved 12,913 authors and generated a total of 12,899 keywords.

#### Evolution of publications

**Figure 1:**
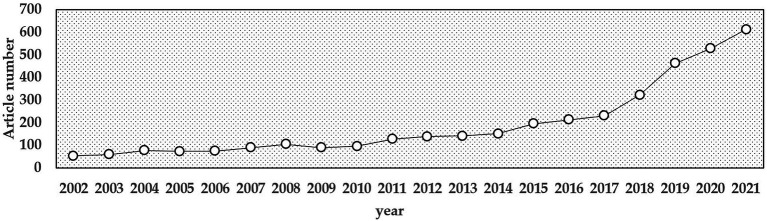
Annual distribution of the number of research publications on agricultural multispectral research in 2002–2021.

The trend of year-on-year growth can be seen in Figure 1, from 54 articles in 2002 to 608 articles in 2021, with an average annual growth rate of 13.5%. The number of publications exceeded 100 for the first time in 2008 and reached 104, then dropped below 100 in the next 2 years. From 2011 the number of publications exceeded 100 again and showed a stable growth trend. From 2002 to 2021, the number of publications increased by 10.25 times. The increasing number of publications year by year indicates that the application of multispectral technology in agriculture is attracting more and more attention.

#### WOS Research Area

Table 1 lists the main agricultural fields of multispectral study from 2002 to 2021, which are remote sensing, imaging science, environmental science, geology, multi-discipline research, agriculture, electronic engineering, etc. Among them, remote sensing is the field with the largest proportion, followed by a variety of subjects, indicating that multispectral research is concerned by multiple fields and disciplines. From the current perspective, the research of multi-discipline integration will become a hot trend in the future about agricultural multispectral research.

**Table 1:** Main categories of multispectral research literature in agriculture from 2002 to 2021.

Subject categories	Number	Ration%
Remote Sensing	1,691	44.11
Imaging Science Photographic Technology	1,430	37.30
Environmental Sciences	1,124	29.32
Geosciences Multidisciplinary	868	22.64
Engineering Electrical Electronic	372	9.70
Geography Physical	295	7.69
Agriculture Multidisciplinary	265	6.91
Agronomy	239	6.23
Plant Sciences	194	5.06

### Analysis of research countries and institutions

**Figure 2:**
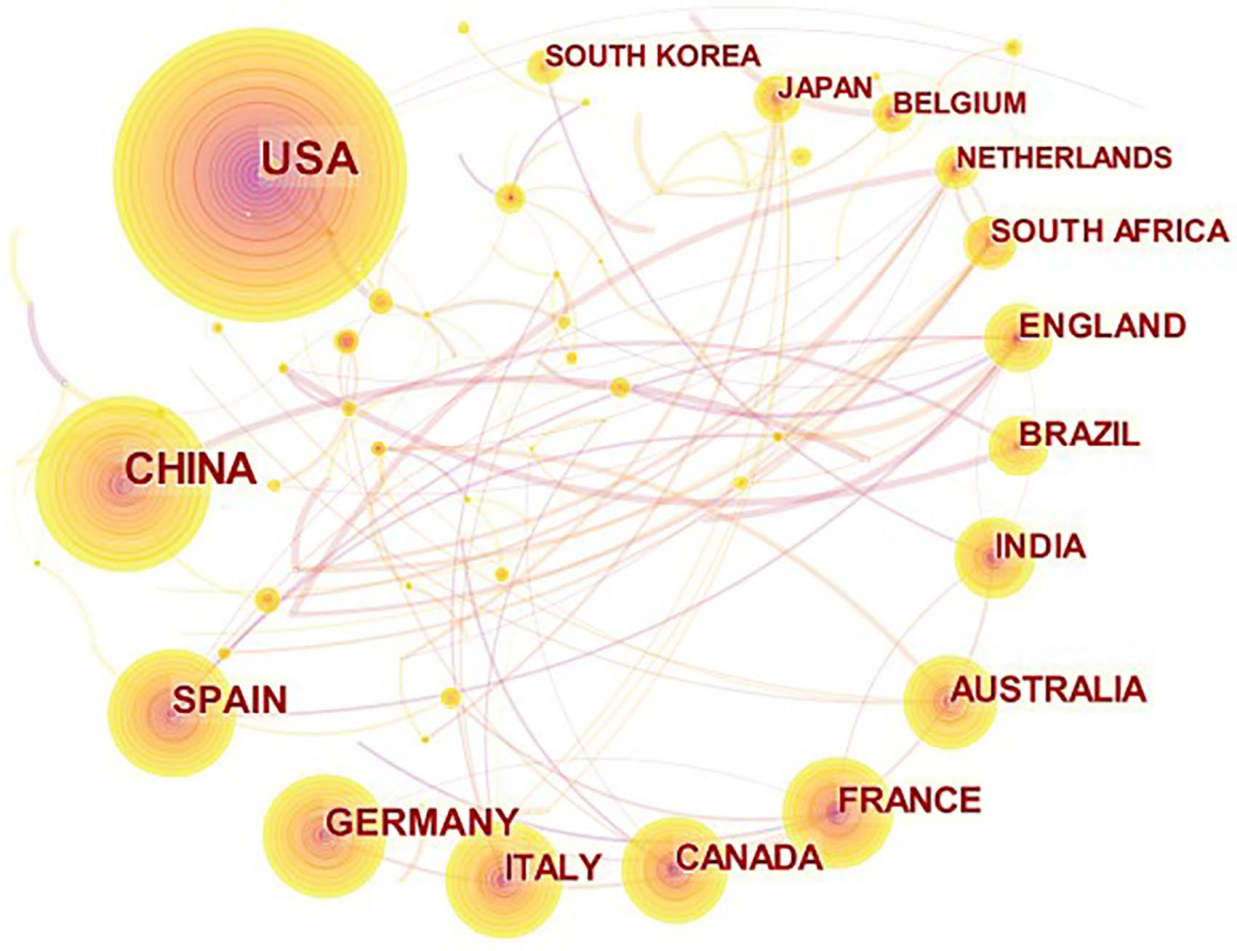
Co-current mapping of research institution collaboration on agricultural multispectral research in 2002–2021. The points to circles represent the individual countries, the size of the graph indicates how much literature comes from each country and how influential it is, and the lines represent how close each country is to other countries.

Analyzing research institutions help understand the publication and collaboration of major institutions. In total, 3,358 institutions are involved in agricultural multispectral research. Results show that the top five institutions with the highest number of publications are: USDA, Chinese Academy of Sciences, NASA, UNIVERSITY OF CALIFORNIA SYSTEM, and CNRS.A total of 746 articles were published by the above five institutions. The top two institutions have significantly more publications than the others, indicating an imbalance between the research publications of influential institutions. From 2002 to 2021, the USDA is the institution with the highest number of publications, with a total of 247 publications, taking the first place. The institutional co-occurrence mapping in Figure 2 was generated based on the analysis.

**Figure 3:**
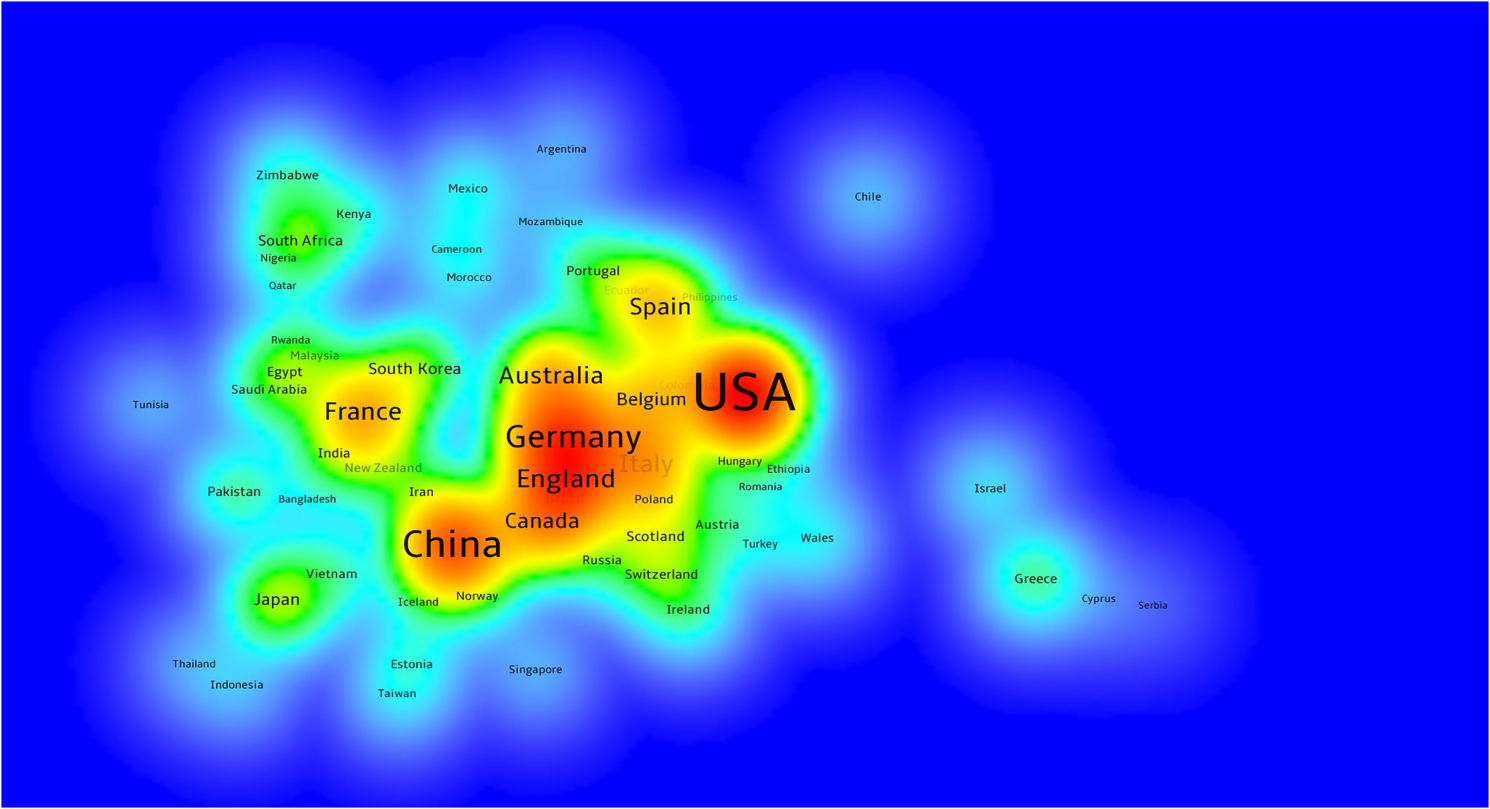
Visualization of agricultural multispectral research in terms of the density of cooperation between countries.

A total of 118 countries are involved in agricultural multispectral research. The density of cooperation among countries is visualized in Figure 3. The research countries analysis shows that the five countries with the highest number of publications are the United States (1,177), China (737), Spain (285), Germany (277), and Italy (231). In addition to the number of publications, centrality is one of the criteria to measure the strength of a country’s research in this field. From the data obtained, the top five countries in terms of centrality are: United States, Germany, Italy, Australia, and England. China and Spain, despite being in the top five in terms of number of publications, are not in the top five in terms of centrality ranking.

### Influential journals

**Figure 4:**
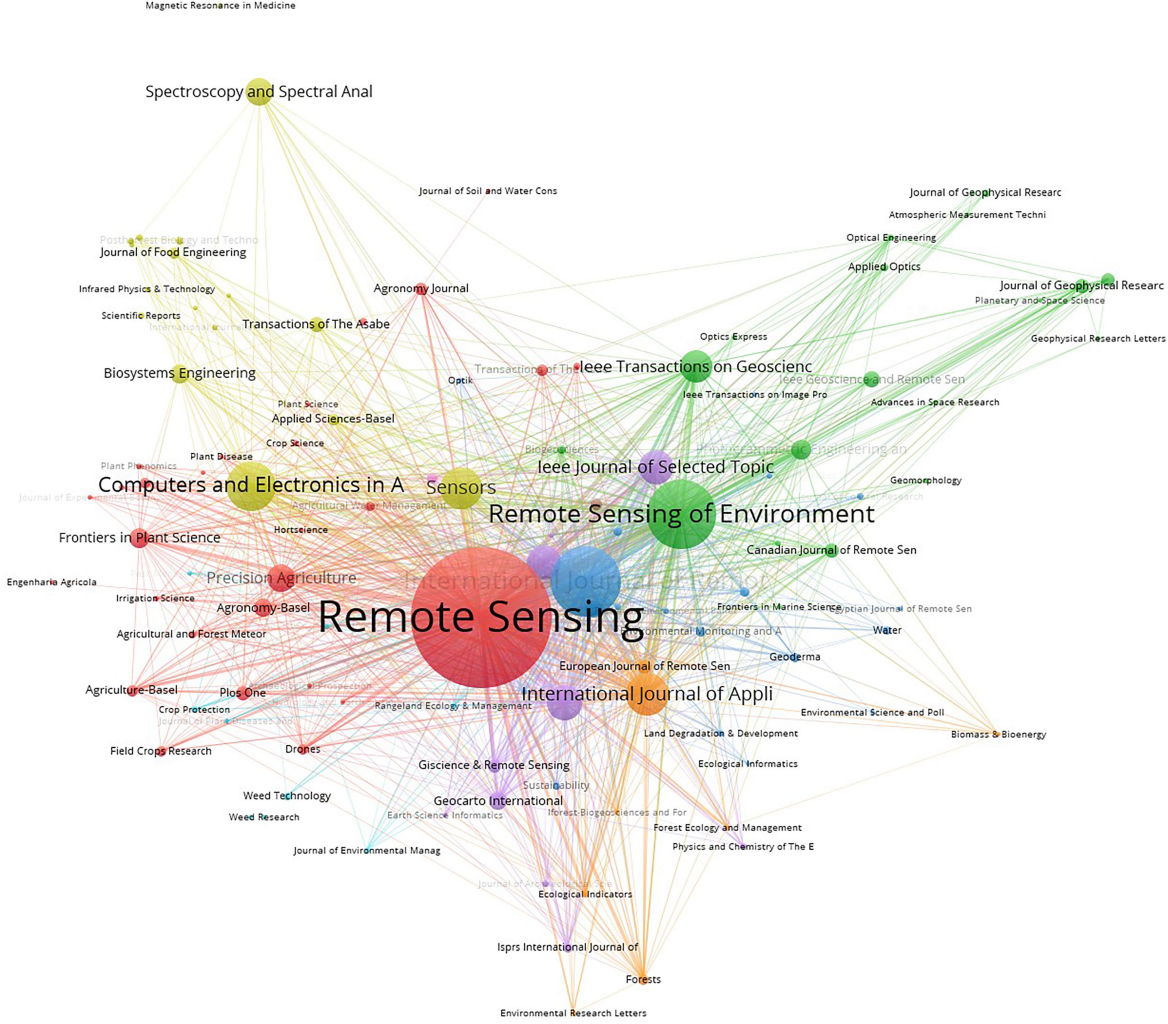
Co-occurrence map of influential journals on agricultural multispectral research in 2002–2021.

Multispectral research in agriculture was published in 702 journals, and the co-occurrence map of journals is shown in Figure 4. The top 20 (2.85%) journals published 2,021 papers, accounting for 52.8% of the total literature. There were 405 journals that published only one paper in agricultural multispectral research, accounting for 57.7% of the total number of journals. There were 200 journals that published 2–5 papers, accounting for 28.5% of the total number of journals. Less than 10 papers were published in 645 journals, representing 91.8% of the total number of journals. The top 3 publishers were Remote sensing (529), International journal of remote sensing (216), and Remote sensing of environment (207). According to this analysis, the agricultural multispectral publications become scattered. Most of the research achievement was published in 12 journals as shown in Table 2. These 12 journals can be considered as the core sources of multispectral research in agriculture, and these journals play an important role as well. Among them, Remote sensing is the most popular journal in the field of agricultural multispectral research with the largest proportion and the fastest growth rate, showing that it has played an important role in promoting multispectral research in agriculture.

**Table 2:** Top 12 journals for local citation ranking of agricultural multispectral studies in 2002–2021.

Source	D	Citations	TS	WL
Remote Sensing	532	7,930	1,831	198
International Journal of Remote Sensing	216	4,934	560	123
Remote Sensing of Environment	207	17,268	1,216	172
Computers and Electronics in Agriculture	134	3,982	495	111
International Journal of Applied earth Observation and Geoinformation Sensors	107	2,766	501	106
Sensors	105	1782	400	92
Isprs Journal of Photogrammetry and Remote Sensing	85	3,293	525	89
Ieee Journal of Elected Topics in Applied Earth Observations and Remote Sensing	84	1743	259	54
Journal of Applied Remote Sensing	80	935	213	63
Ieee Transactions on Image Processing	79	4,137	309	75
Precision Agriculture	62	1952	275	29
Spectroscopy and Spectral Analysis	60	174	34	18

D, documents; TS, Total link strength; WL, weight links.

### Analysis of author groups

**Figure 5:**
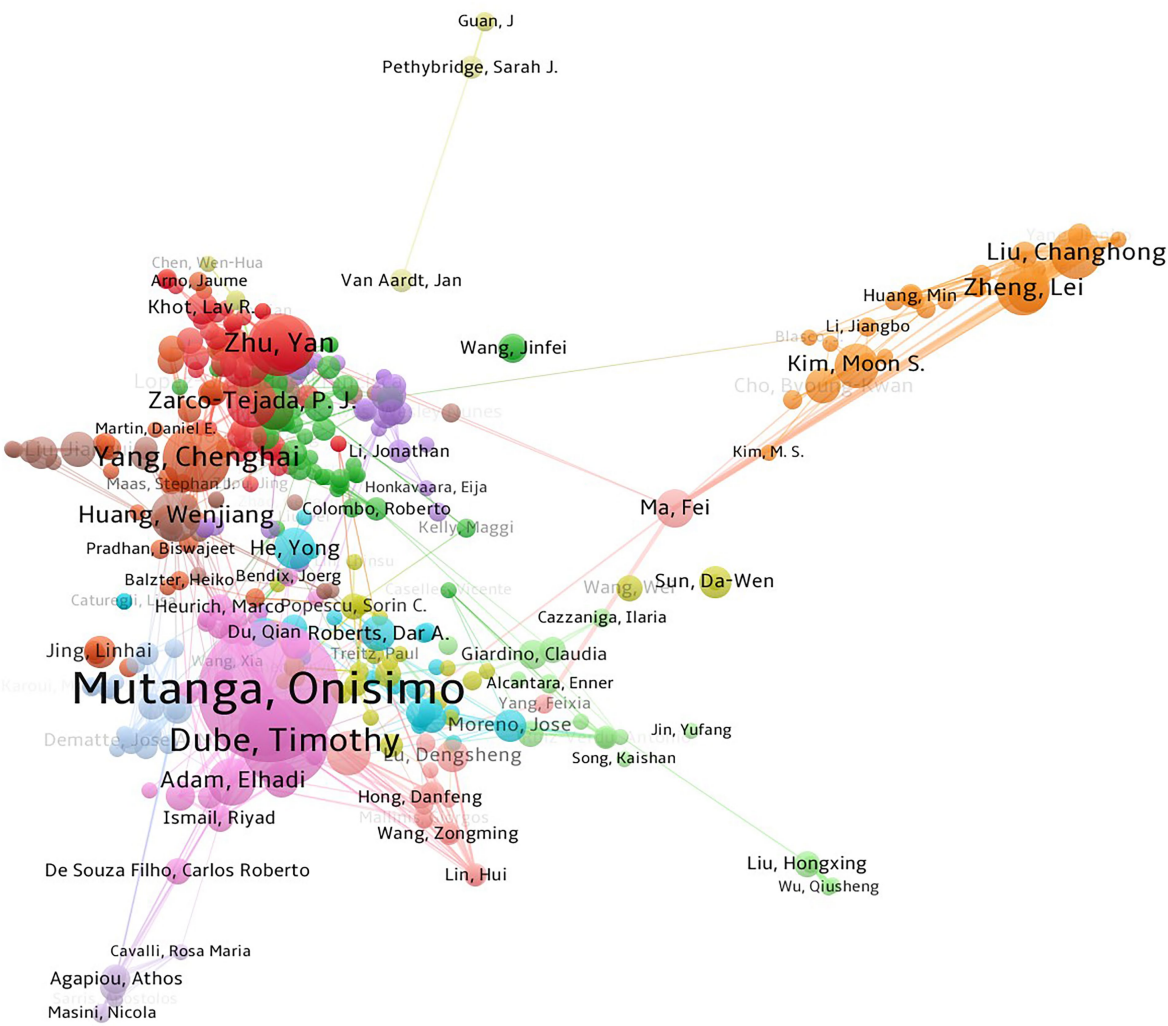
Co-occurrence map of agricultural multispectral researcher collaboration in 2002–2021.

The collaborative network of authors enables the analysis of the core authors and collaborations within the field of agricultural multispectral study. The analysis of core authors and their collaborative relationships was performed by VOSviewer to generate a co-occurrence map of authors, as shown in Figure 5. Results show that the top 5 authors with the highest number of published papers are Onisimo Mutanga (52), Timothy Dube (25), Yu Zhang (24), James F. Bell (22), and Jeffrey R. Johnson (22). Zarco-Tejada, P. J. has the most cited papers with an average of 81.9 citations per paper. Authors with more than four publications in the field of study were considered core authors according to its definition, of which there are 675 core authors. The top 10 core authors are shown in Table 3. There are 3,830 papers that involve 12,913 authors, with an average of 0.29 papers per author and 3.37 authors per paper. This also indicates that multispectral research in agriculture is a multi-author collaborative field.

**Table 3:** Top 10 authors ranked by total literature on agricultural multispectral research in 2002–2021.

Authors	Dc	Ct	TLS	Institutions	Countries
Onisimo Mutanga	52	1,672	813	University of KwaZulu-Natal	South Africa
Timothy Dube	30	591	427	University of Western Cape	South Africa
Yu Zhang	23	563	320	Chinese Academy of Sciences	China
James F. Bell	22	396	199	Arizona State University	United States
Jeffrey R. Johnson	22	1,228	260	U.S. Geological Survey	United States
Chenghai Yang	22	563	209	USDA-Agricultural Research Service	United States
Moon S. Kim	21	272	68	USDA-Agricultural Research Service	United States
Lopez-granados F	21	891	163	Spanish National Research Council	Spain
Lei Zheng	21	291	568	Hefei University of Technology	United States
Yan Zhu	21	396	199	Nanjing Agricultural University	China

Dc, Documents; Ct, Citations; TLS, Total link strength; JCICTA, Jiangsu Collaborative Innovation Center for the Technology and Application of Internet of Things; UKSARC, USDA-ARS Kika de la Garza Subtropical Agricultural Research Center.

### Keyword analysis

**Figure 6:**
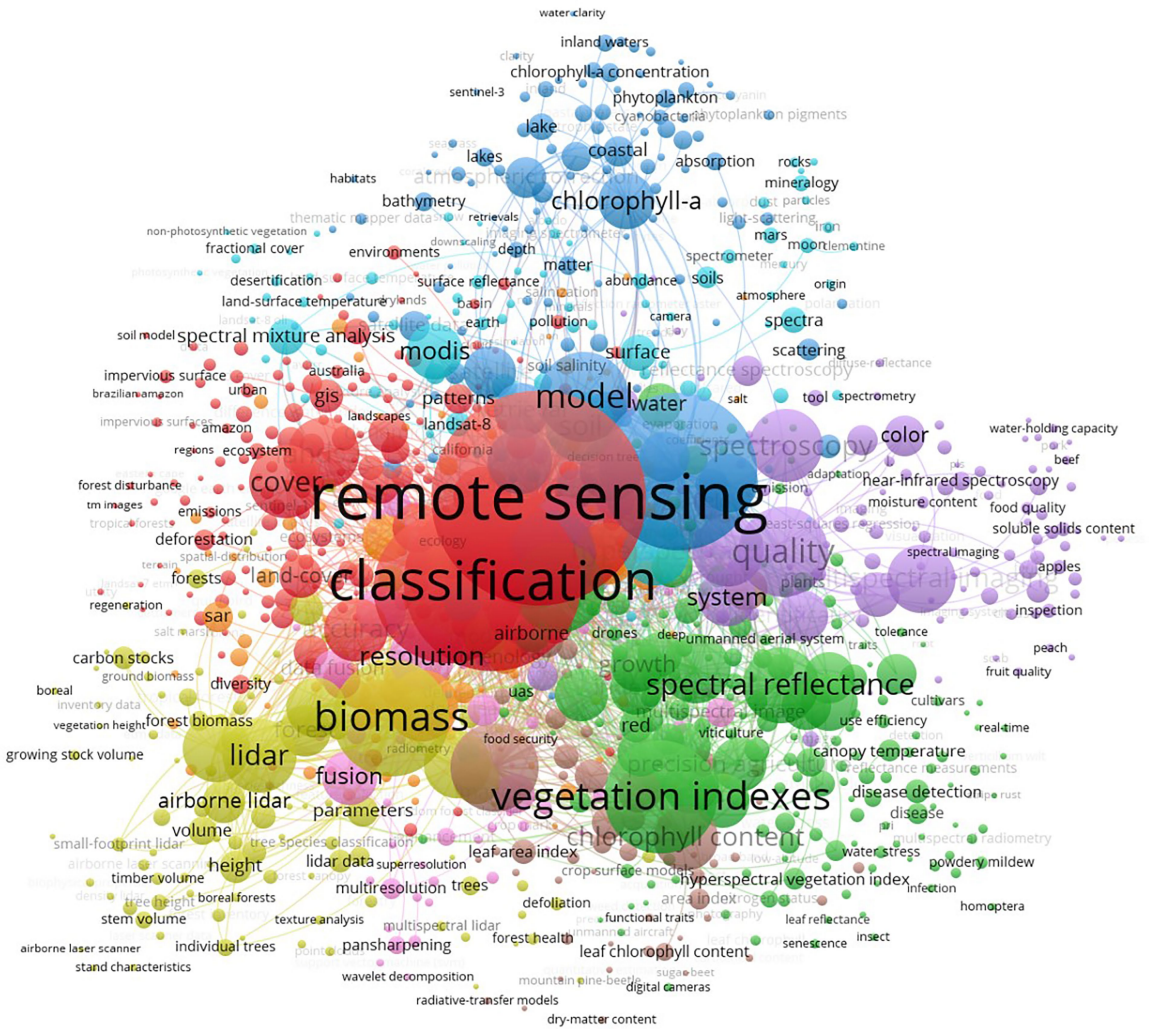
Network of keywords based on the co-occurrence method on agricultural multispectral research in 2002–2021.

A total of 12,899 keywords were detected in 3,830 publications from 2002 to 2021 through software analysis. A total of nine clusters were generated by co-occurrence analysis. The clusters, based on the relationship between the weight of link attributes under different keywords and the strength of total links, are shown in Figure 6. The top 20 keywords in 3,212 publications were ranked by frequency, as shown in Table 4.

**Table 4:** Top 20 keywords ranked by frequency on agricultural multispectral research in 2002–2021.

P	Keyword	N	C	L	TS	P	Keyword	N	C	L	TS
1	Remote sensing	532	1	1,267	4,212	11	NDVI	163	2	681	1,493
2	Classification	416	1	1,075	3,475	12	Lidar	160	4	570	1,339
3	Reflectance	374	3	1,062	3,188	13	Spectral reflectance	150	2	621	1,501
4	Vegetation	334	2	991	2,965	14	Uav	149	2	541	1,239
5	Biomass	302	4	791	2,288	15	Soil	145	6	544	1,324
6	Vegetation indexes	251	2	741	2,230	16	Yield	141	2	515	974
7	Leaf-area index	191	8	662	1,511	17	Cover	139	1	542	1,143
8	Imagery	174	5	654	1,446	18	Spectroscopy	137	5	564	1,140
9	Multispectral	171	4	584	1,325	19	Prediction	135	5	538	1,276
10	Model	165	3	605	1,615	20	Precision agriculture	132	5	518	1,137

P, position; N, number of articles; C, Cluster; L, Weight Links; TS, Total link strength.

Figure 6 shows the keyword network presented by the co-occurrence method on agricultural multispectral research in 2002–2021. The keywords are divided into nine clusters and each cluster is identified by a different color. The thickness and number of connecting lines between different clusters indicate the closeness of the connection between clusters. These nine clusters are cluster 1, red: remote sensing; cluster 2, green: vegetation indexes; cluster 3, cyan: reflectance; cluster 4, yellow: biomass; cluster 5, purple: quality; cluster 6, blue: soil; cluster 7, orange: random forest; cluster 8, brown: leaf-area index; cluster 9, pink: resolution. From the top 20 keywords given on Table 4, cluster 1 remote sensing, cluster 2 vegetation indexes, cluster 3 reflectance, cluster 4 biomass, cluster 5 quality account for the most weight, by clustering with the four keywords that account for the most, we can know the current research hotspots.

By keyword clustering found clustering 1 remote sensing in the field of agricultural multispectral is the most dominant research area, remote sensing technology is generally considered as one of the most important technologies for precision agriculture, and the development of technologies such as imaging of spectral information and multi-directional optical detection has improved the timeliness and operability of remote sensing technology (Tsouros et al., 2019). At present, remote sensing in the field of agriculture mainly focuses on: crop classification, crop coverage, and precise identification. Classification of weeds and crops in the field and accurate management of weeds, crop cover and vegetation coverage in agricultural fields, accurate identification of biomass and trace element content are the current research priorities in remote sensing (Huang et al., 2018; Näsi et al., 2018; Memon et al., 2019; Wijesingha et al., 2021). Remote sensing has made a great contribution to the development of precision agriculture.

Keyword clustering 2 led by vegetation indexes is likewise a current research hotspot in multispectral in agriculture, vegetation indexes have been widely used to qualitatively and quantitatively evaluate the information produced by vegetation, and vegetation indexes have been applied in a particularly wide range of applications, in which yield prediction, spectral reflection studies, vegetation growth, and vegetation canopy information extraction are hot research directions (Zhou et al., 2017; Marston et al., 2020; Peng et al., 2021; Zhu et al., 2021). Among these research directions, accurate yield prediction of food crops, application research of specific wavelength bands, soil drought and salinity, foliar index, and crop pest monitoring are the focus of research (Farrell et al., 2018; Mazzia et al., 2020; Zhu et al., 2021), and a significant part of these studies use uavs as the main application platform, and the combination of UAVs and multispectral further promotes the development of vegetation indices in agriculture.

keyword clustering 3 reflectance is based on spectral reflectance information to carry out various studies in which the most important thing is to use reflectance information to construct models through which specific problems can be solved. For example, the simple algorithm yield model was used to estimate the foliar index by combining the light energy efficiency and leaf function of the crop (Peng et al., 2021) the SWAP-WOFOST model was used to predict the growth of sugarcane (Hu et al., 2019) and the improved casa model was used to map the crop biomass (Fang et al., 2021). The application of various models helped us to provide a great role in rapid crop monitoring as well as crop yield assessment.

Keywords clustering 4 biomass: Biomass is a common crop parameter based on remote sensing and with the rapid development of remote sensing technology biomass detection techniques have advanced tremendously with the rapid development of precision agriculture from 1980 to 2021. The rapid development of UAV technology, lightweight multispectral, and hyperspectral equipment has provided new tools for biomass detection and during these decades most of the studies on crop parameters were conducted based on spectral information and with the addition of 3D information technology the interplay of the two is a new progress in the detection of crop parameters (Candiago et al., 2015; Näsi et al., 2018; Zhu et al., 2019a; Fei et al., 2021; Jayakumari et al., 2021; Li et al., 2021; Yu et al., 2021).

Keywords clustering 5 quality: Improving the quality of production is one of the goals of agriculture and the development of multispectral technologies in improving the quality of production provides scientific tools to achieve the goal of high-quality agricultural development and the rapid development of UAVs with light and portable sensors capable of capturing multiple spectral images and new image processing methods have promoted high quality agricultural production (Messina et al., 2021). For example, six-band multispectral sensors and accurate orthorectified impact processing methods can improve spatial accuracy and can provide guidance for subsequent research (Mesas-Carrascosa et al., 2017); the use of deep learning methods to segment UAV images a new image alignment method that enables the fusion of information from two different sensors and improves detection accuracy (Kerkech et al., 2020).

### Theme term analysis

#### Co-occurrence analysis of theme term

**Figure 7:**
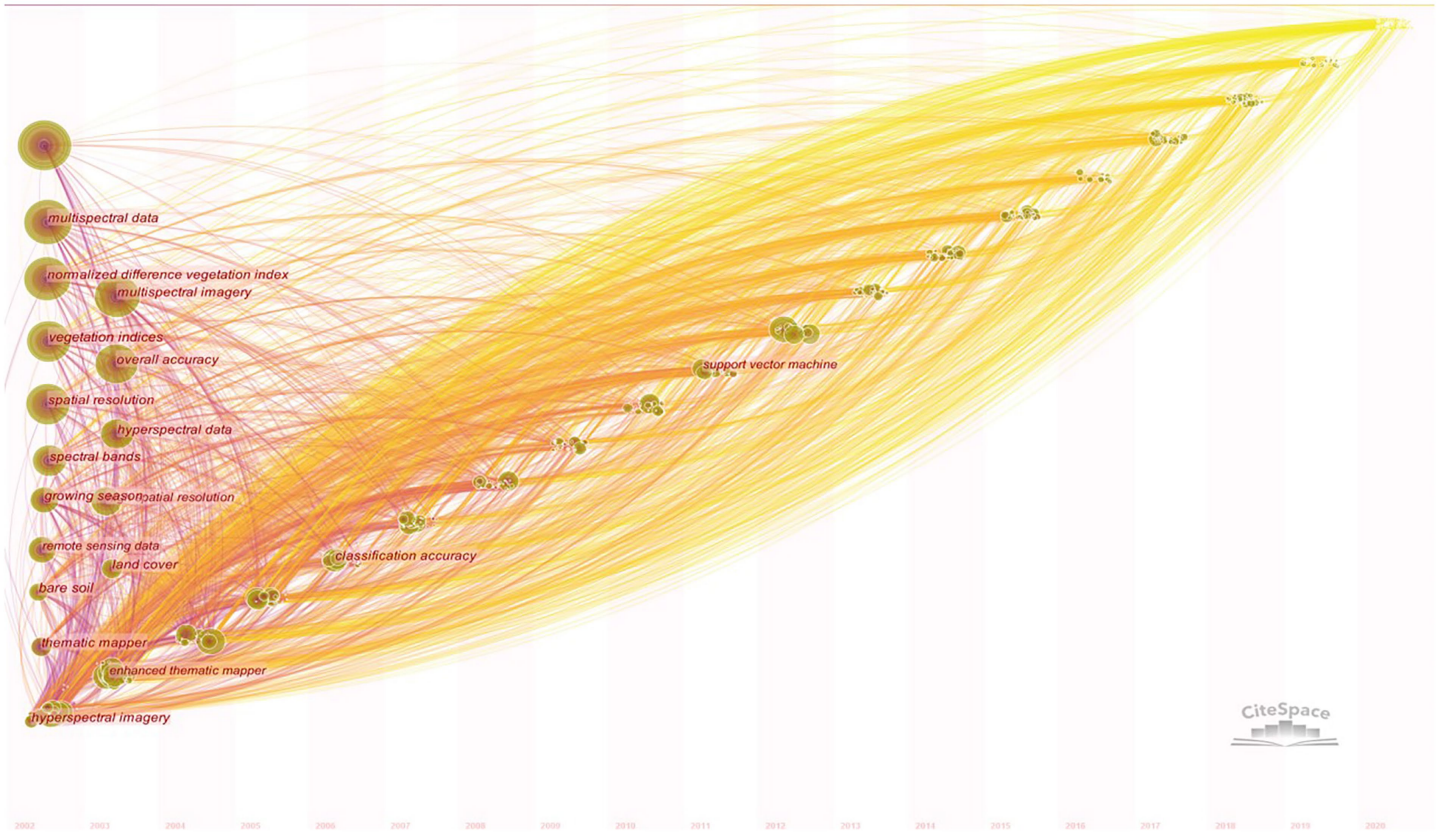
Co-occurrence time zone map of theme terms on agricultural multispectral research in 2002–2021. The horizontal axis represents the year, each node represents a topic, and the size of each node represents frequency of occurrence. The lines between each node represent connections to other topics. The circles in the vertical axis represent topics in the multispectral area of agriculture. The size of the circles represents the magnitude of the heat. The years with more topics were arranged in order from the largest to the smallest on the vertical axis.

The theme term was analyzed by CiteSpace. Noun terms were extracted from the titles, author keywords, and system supplementary keywords of the dataset, and the top appearing terms were selected to generate a network time zone map (Figure 7). The time zone map collects the first occurrence of theme terms in the same year and the same time zone, which shows the evolution of the knowledge domain in the time dimension more clearly. Multispectral image, vegetation indices, normalized difference vegetation index (NDVI), the multispectral data, Unmanned aerial vehicle, overall accuracy, square error, spectral bands, random forest, high spatial resolution, hyperspectral data, remote sensing data, spatial distribution, growing season, etc., should be considered as the hot content of multispectral research in the field of agriculture in these years.

#### Burst Analysis of Theme Term

The theme terms with relatively high salience were analyzed by CiteSpace’s burst detection algorithm in order to reflect the research trend and dig out the research hotspots, which are characterized by high frequency of changes within a certain phase in the software. The nodes that show up in red in Figure 7 indicate burst. Since there are relatively more red nodes with prominence from 2002 to 2021, the red nodes with prominence in the 6 years from 2015 to 2021 were selected as shown in Table 5. Satellite data, climate change, forest inventory, standard deviation, growing satellite data, climate change, forest inventory, standard deviation, growing seasons, plant height, sentinel-2 data, unmanned aerial, environmental condition, crop water stress index, point clouds, crop yield, etc., indicate that the recent years of multispectral research in agriculture are based on the above series of themes. The above-mentioned thematic terms have been used as a research method in the field of agriculture. From 2002 to 2010, burst themes included canopy reflectance, spectral mixture analysis, reflectance data, airborne multispectral imagery, aerial photography, and multispectral analysis. Burst themes in 2011–2021 associated with multispectral satellite imagery, remote sensing technique, high resolution, water quality parameter, partial least squares, crop water stress index, and biomass estimation, which indicate that the research and application of vegetation indices and algorithms were the main focus at this phase. Among them, vegetation index is the most researched part of multispectral in the field of agriculture.

**Table 5:** Burst theme terms on agricultural multispectral study in 2015–2021.

Term	Strength	Begin	End	2015–2021
Satellite data	3.81	2015	2017	▂▂▂▂▂▂▂▂▂▂▂▂▂▃▃▃▂▂▂
Climate change	3.44	2015	2017	▂▂▂▂▂▂▂▂▂▂▂▂▂▃▃▃▂▂▂
Forest inventory	3.15	2015	2018	▂▂▂▂▂▂▂▂▂▂▂▂▂▃▃▃▃▂▂
Spectral feature	5.07	2016	2018	▂▂▂▂▂▂▂▂▂▂▂▂▂▂▃▃▃▂▂
Hyperspectral image	4.73	2016	2017	▂▂▂▂▂▂▂▂▂▂▂▂▂▂▃▃▂▂▂
Standard deviation	3.77	2016	2021	▂▂▂▂▂▂▂▂▂▂▂▂▂▂▃▃▃▃▃
Growing seasons	3.69	2016	2021	▂▂▂▂▂▂▂▂▂▂▂▂▂▂▃▃▃▃▃
Soil property	5.91	2017	2018	▂▂▂▂▂▂▂▂▂▂▂▂▂▂▂▃▃▂▂
Optimal wavelengths	4.6	2017	2021	▂▂▂▂▂▂▂▂▂▂▂▂▂▂▂▃▃▃▃
Plant height	3.1	2017	2021	▂▂▂▂▂▂▂▂▂▂▂▂▂▂▂▃▃▃▃
Sentinel-2 data	8.1	2018	2021	▂▂▂▂▂▂▂▂▂▂▂▂▂▂▂▂▃▃▃
Unmanned aerial system	6.02	2018	2021	▂▂▂▂▂▂▂▂▂▂▂▂▂▂▂▂▃▃▃
RGB image	5.71	2018	2021	▂▂▂▂▂▂▂▂▂▂▂▂▂▂▂▂▃▃▃
Field scale	4.82	2018	2021	▂▂▂▂▂▂▂▂▂▂▂▂▂▂▂▂▃▃▃
Environmental condition	4.02	2018	2021	▂▂▂▂▂▂▂▂▂▂▂▂▂▂▂▂▃▃▃
Crop water stress index	4.02	2018	2021	▂▂▂▂▂▂▂▂▂▂▂▂▂▂▂▂▃▃▃
Point clouds	3.62	2018	2021	▂▂▂▂▂▂▂▂▂▂▂▂▂▂▂▂▃▃▃
Digital surface model	3.19	2018	2021	▂▂▂▂▂▂▂▂▂▂▂▂▂▂▂▂▃▃▃
Crop yield	3.19	2018	2021	▂▂▂▂▂▂▂▂▂▂▂▂▂▂▂▂▃▃▃

### Research frontiers

**Figure 8:**
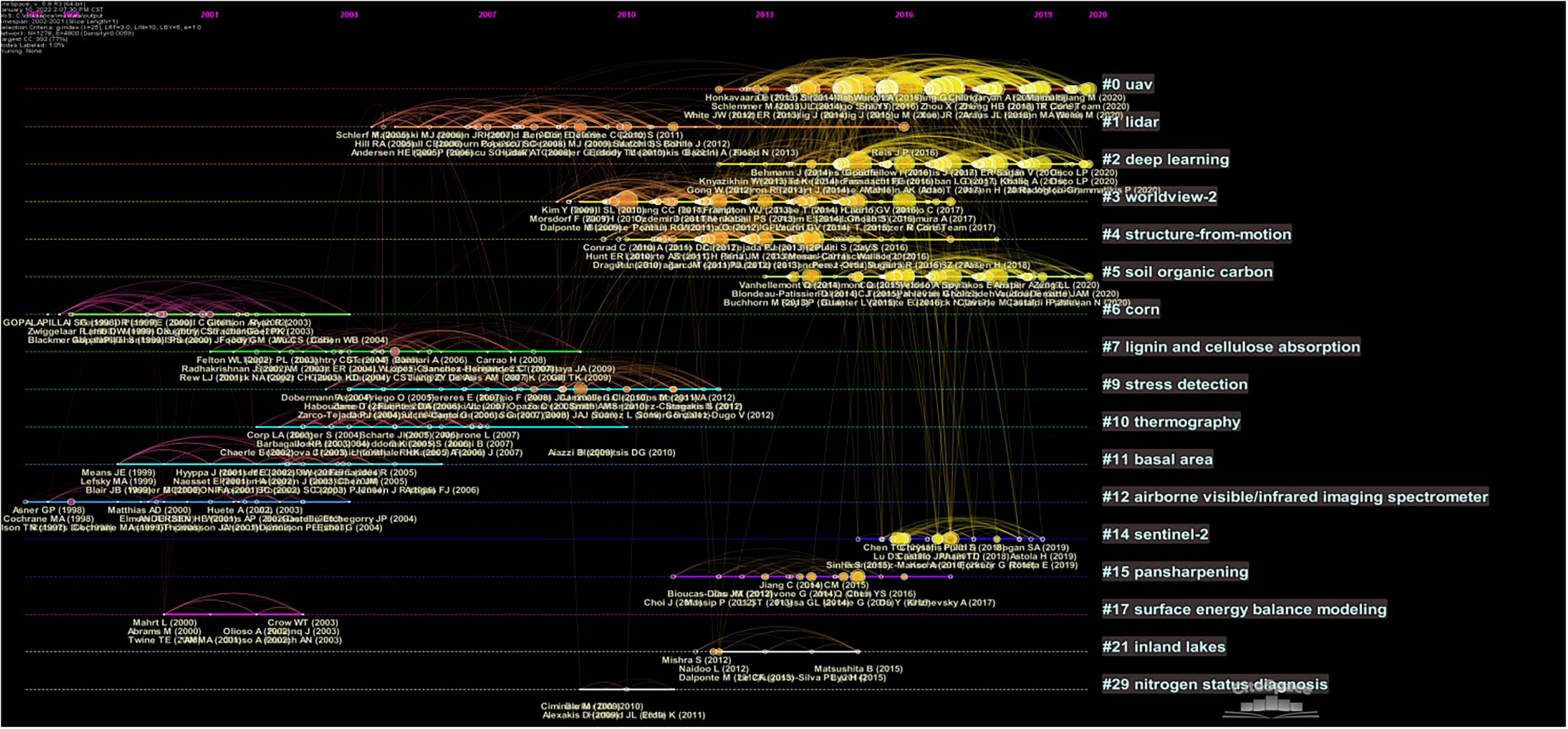
Co-citation timeline mapping of publications on agricultural multispectral research in 2002–2021. The horizontal axis represents the year, each node represents a popular cited reference, and the size of each node is proportional to its citation frequency. The line between each node represents the time evolution of the cited literature, and the thickness of the line represents the co-citation intensity.

Agricultural multispectral research analysis selects the reference node and generates the cited literature analysis graph by CiteSpace. Clusters were formed by selecting keywords, and then a total of 17 clusters were generated by LLR algorithm. Each of these clusters represents the activity of its future direction, as shown in Figure 8. The denser and more active the clusters in the graph, the more they represent the current research frontier. The module values of the 17 clusters in Figure 8 are *Q* = 0.8208 and *S* = 0.917. The module values and the average profile values are indicators provided by CiteSpace based on the network structure and the clarity of the clusters, which are used to measure the clustering effect of the map. *Q* > 0.3 indicates that the clustering is significant, and *S* > 0.7 indicates that the clustering is highly convincing. Generally, the clustering above 0.5 can be considered reasonable. Therefore, the present clustering is reasonable and the structure is significant.

The color curves in the figure indicate co-citation links. More connected lines between clusters indicate a strong correlation between clusters. Large nodes indicate that they are worth exploring because they contain important cases that are overcited or mentioned. Nodes that are still active represent cutting-edge directions, scientific themes and novel trends in agricultural multispectral research field. According to the Figure 8, the largest cluster is #0 (vegetation index), located at the top of the image. The duration varies between clusters, with some lasting more than a decade and others having a shorter life span. The four clusters with higher activity and frequency were selected for further analysis.

The largest cluster #0 (UAV) contains 175 reference points between 2012 and 2021. The average reference year is 2016 and the average profile value *S* = 0.855, which was well visualized. This cluster is a corresponding study for vegetation indices in land cover, vegetation classification, crop yield estimation, drought monitoring, and environmental change using UAV remote sensing as the main application platform. UAV is an important vehicle for acquiring a variety of remote sensing data accurately, flexibly and efficiently in the low-altitude field, and UAV remote sensing is now very commonly used in precision agriculture, while UAV-based IoT technology is considered as the future of remote sensing in precision agriculture. Vegetation indices are formed by combining different bands of the spectrum according to the spectral characteristics of vegetation. More than 40 kinds of vegetation indices are available and widely used in the field of agricultural production. The most cited one in this cluster is Bendig et al. (2015), who used vegetation indices and plant height information to estimate summer barley biomass and verified the potential of visible bands to predict biomass. Candiago et al. (2015) demonstrated the great potential of UAVs in the multispectral field by monitoring vegetation indices with UAVs carrying imaging equipment. Zhou et al. (2017) verified that red-edge and infrared bands were more effective in predicting rice yield and foliar index based on a UAV-mounted multispectral photography platform, demonstrating the reliability of the platform for rice yield and growth estimation and identifying the most contraindicated virtual instrument for rice yield. Zaman-Allah et al. (2015) used a multispectral imaging sensor-mounted UAV platform to measure the N content in soil and derive crop performance indices for low N stress in maize fields, showing that the platform is effective in assessing field variability and crop performance. Duan et al. (2017) applied UAV based multispectral camera to detect NDVI indices in wheat field during the growing season through a high-throughput phenotyping platform, showing that NDVI before and after flowering had a strong correlation with yield. From the clustering timeline, the cluster is still highly dynamic until 2021, and UAV remote sensing is still a hot spot for current research.

The average citation time for cluster #2 is 2016. This cluster focuses on the clustering of remote sensing-based algorithms, which summarizes a review of multispectral remote sensing and case studies of algorithm applications. Currently algorithms for remotely sensed vegetation indices is effective and convenient, which have been applied for vegetation cover and growth dynamics research with wide application of UAVs (Xue and Su, 2017). Torres-Sánchez et al. (2015a) newly developed a threshold segmentation OBIA algorithm by UAV images on the Otsu-based method. This algorithm was applied to the Excess Green Index (ExG) and Normalized Vegetation Index (NDVI). Torres-Sánchez et al. (2015b) proposed a method to calculate the 3D geometric features of individual trees and rows with an accuracy of 97% for area quantification with UAVs. Mathews and Jensen (2013) acquired UAV images to collect Leaf Area Index (LAI) of visualized and quantified vineyard canopy through a motion point cloud computer technique. Albetis et al. (2017) proposed a UAV-based images and operational flavescence dorée mapping technique for grape diseases detection. The duration of this clustering is 2012–2021 and, as with the vegetation index, machine learning is also a current hotspot. Machine learning has becomes a hot spot thanks to the development of artificial intelligence, which improves efficiency by combining various algorithms. Integration of machine learning and vegetation indices plays an important role in the field of agricultural multispectral research.

Cluster #3 is well visualized with a mean citation time of 2012 and a mean profile of *S* = 0.897. The clustering is mainly the analysis of ground information using high-altitude images from satellite multispectral remote sensing. Nowadays, more and more UAVs are joining the application of remote sensing mapping, but satellite remote sensing is still the most used method in high-altitude remote sensing. Satellite remote sensing has the characteristics of high point of view, wide field of view, and continuous and fast data collection. It has a broad application prospect in land resources, water resources survey, farming estimation, etc. Dube and Mutanga (2015) applied the medium-resolution multi-spectral Landsat 8 to analyze above-ground biomass in forest plantations. His study concluded that the data provided by satellite could be a more effective data source for analyzing above-ground biomass and spectral vegetation indices, demonstrating the potential and advantages of this Landsat dataset. Immitzer et al. (2016) adopted S2 satellites to map summer and winter crops and different deciduous and tree species to confirm the capability of S2 data for land cover mapping and the high value of red-edge and short-wave infrared bands for vegetation mapping. Clevers and Gitelson (2013) estimated chlorophyll and nitrogen content of crops and grass based on red-edge band remote sensing on Sentinel-2 and-3 satellites, confirming the importance of the red-edge band on satellites for agricultural applications. Ramoelo et al. (2015) used Worldview-2 satellite to monitor leaf nitrogen content and above-ground biomass, demonstrating the importance of high-altitude resolution and the red-edge band in rangeland assessment and detection. Mutanga et al. (2012) obtained red-edge-band images from Worldview-2 satellite and applied random forest regression algorithm to predict biomass in wetland areas. The duration of this clustering was 2009–2017, which indicates that multispectral research in terms of satellites is no longer a hotspot for research at present. The current direction of research has moved toward multispectral remote sensing research represented by UAVs.

The average citation time for cluster #4 is 2012. The main research in this cluster is summarized for aerial detection. In addition, this cluster is a multispectral research mainly by UAVs. The main research in this cluster is remote sensing mapping of field vegetation by multispectral information, and building higher accuracy classification models, including research on weed identification and yield estimation in crop fields. Yu et al. (2016) adopted UAV platform to obtain multispectral data for soybean breeding, which led to significant improvement in yield estimation models through machine learning. Maimaitijiang et al. (2017) applied UAV multisensor data fusion to extract soybean plant phenotypes and developed a model for extracting plant phenotypes, demonstrating that low-cost multisensors can provide accurate data. Veeranampalayam Sivakumar et al. (2020) conducted experiments on weed identification with low altitude UAV image of soybean field, verifying the accuracy of the Faster RCNN model and fully affirming the importance of the model. Marston et al. (2020) conducted soybean aphid experiments with UAVs and found that NIR reflections are sensitive for aphids detection. The duration of clustering #4 was from 2010 to 2018, and the research on re-clustering provided a basis and reference for the subsequent development of multispectral technology in agriculture in terms of precision management of farmland, as well as crop identification. Along with the rise of vegetation indices as well as machine science, remote sensing mapping classification is gradually approaching this aspect.

In addition, based on the ranking of the burst literature according to the intensity, the top 15 articles were selected from total 162 strongly burst literatures, as shown in Table 6. From the content presented in these 15 literatures, the results are roughly the same as those of the above cluster analysis, with the research frontier trends focusing on the application and expansion of the UAV platform in various aspects, as well as various studies for vegetation indices. Data acquisition mostly relies on multispectral sensors carried by UAVs (Zhang and Kovacs, 2012) and to a lesser extent on satellites (Clevers and Gitelson, 2013), with UAVs gradually occupying the mainstream with their low-cost and flexibility advantages. Moreover, most of the studies are on crop parameters such as biomass (Bendig et al., 2015), species classification (Ke et al., 2010), plant phenotypes (Araus and Cairns, 2014), etc. Machine learning (Mountrakis et al., 2011) also plays a big role in this. The development of new and emerging technologies has played a great role in the development of agriculture.

**Table 6:** Top 15 highest prominence of cited references.

Begin	End	Strength	Year	References	2002–2021
2011	2015	15.5696	2010	Blaschke, 2010, ISPRS J Photogramm, V65, P2	▂▂▂▂▂▂▂▂▂▃▃▃▃▃▂▂▂▂▂
2013	2017	13.1026	2012	Zarco-Tejada PJ, 2012, Remote Sens Environ, V117, P322	▂▂▂▂▂▂▂▂▂▂▂▃▃▃▃▃▂▂▂
2017	2018	12.5081	2013	Clevers JGPW, 2013, Int J Appl Earth Obs, V23, P344	▂▂▂▂▂▂▂▂▂▂▂▂▂▂▂▃▃▂▂
2015	2018	12.1329	2013	Mulla DJ, 2013, Biosyst Eng, V114, P358	▂▂▂▂▂▂▂▂▂▂▂▂▂▃▃▃▃▂▂
2014	2017	11.6238	2012	Mutanga O, 2012, Int J Appl Earth Obs, V18, P399	▂▂▂▂▂▂▂▂▂▂▂▂▃▃▃▃▂▂▂
2012	2014	11.4153	2009	Berni JAJ, 2009, IEEE T Geosci Remote, V47, P722	▂▂▂▂▂▂▂▂▂▂▃▃▃▂▂▂▂▂▂
2017	2021	10.5978	2014	Bendig J, 2014, Remote Sens, V6, P10395	▂▂▂▂▂▂▂▂▂▂▂▂▂▂▂▃▃▃▃
2015	2017	9.5025	2012	Zhang CH, 2012, Precis Agric, V13, P693	▂▂▂▂▂▂▂▂▂▂▂▂▂▃▃▃▂▂▂
2006	2017	9.3241	2012	Berner L T, 2012, Biogeosciences, V9, P3943	▂▂▂▂▂▂▂▂▂▂▃▃▃▃▃▃▂▂▂
2011	2014	9.2223	2009	Chander G, 2009, Remote Sens Environ, V113, P893	▂▂▂▂▂▂▂▂▂▃▃▃▃▂▂▂▂▂▂
2018	2021	8.3433	2015	Bendig J, 2015, Int J Appl Earth Obs, V39, P79	▂▂▂▂▂▂▂▂▂▂▂▂▂▂▂▂▃▃▃
2012	2015	8.1168	2010	KeYH, 2010, Remote Sens Environ, V114, P1141	▂▂▂▂▂▂▂▂▂▂▃▃▃▃▂▂▂▂▂
2017	2021	7.7503	2014	Torres-Sanchez J, 2014, Comput Electron Agr, V103, P104	▂▂▂▂▂▂▂▂▂▂▂▂▂▂▂▃▃▃▃
2016	2021	7.1715	2014	Araus JL, 2014, Trends Plant Sci, V19, P52	▂▂▂▂▂▂▂▂▂▂▂▂▂▂▃▃▃▃▃
2012	2016	7.1502	2011	Mountrakis G, 2011, ISPRS J Photogramm, V66, P247	▂▂▂▂▂▂▂▂▂▂▃▃▃▃▃▂▂▂▂

## Discussion

Based on the analysis of the publications searched, it is known that the current research on multispectral in agriculture is mainly focused on vegetation index (Chang et al., 2020; Kim et al., 2020; Mazzia et al., 2020), land cover (Laamrani et al., 2020), vegetation classification (Gibson et al., 2004), crop estimation (Zhou et al., 2017), drought monitoring (Periasamy and Shanmugam, 2016), and environmental change (Brook et al., 2020). Among them, the studies related to vegetation indices involves the most papers. Most of the current monitoring of crops uses remote sensing technology and ground data in conjunction with each other to invert the biological indicators of crops, such as normalized vegetation index and biomass (Li et al., 2020a). Vegetation indices are the key to qualitative and quantitative assessment of vegetation, and vegetation indices have been widely applied for crop monitoring. Research related to vegetation indices is also a hot area of research at present and also a hot area of research in the future. Vegetation indices can be divided into linear combinations of bands or original band ratio design (RVI; Lee et al., 2020), improvement of original indices by physical and mathematical methods (with universal applicability; Zhu et al., 2019b), and indices born on the basis of remote sensing technology for hyperspectral and thermal infrared remote sensing (with difficulty in data acquisition and difficult to be promoted and developed; Berni et al., 2009). The early studies of vegetation indices were on chlorophyll and nitrogen measurements, and plant data were obtained by tgi indices in conjunction with related factors (Hunt et al., 2013). Many current studies address NDVI indices and are interwoven with various indices such as NDRE and NGRDI (Hassan et al., 2019). The best proof of the application of remote sensing in agricultural monitoring was demonstrated in crop yield estimation (Maimaitijiang et al., 2020). Combined with the 2021 research literature, there is now a shift from large scale and large area detection of satellite data to specific range detection with further improvement in accuracy and precision.

Before the rise of UAVs, land satellites were primary means of acquiring multispectral data. Satellites equipped with multispectral instruments have the capability to detect the full spectral band from visible to thermal infrared. Such satellites are used in land resources (Zanardo et al., 2016), environmental monitoring (Brook et al., 2020), etc. by higher applications. And in recent years the emergence of unmanned aircraft remote sensing detection gradually occupy the mainstream, but from 2002 to the present satellites still mainly undertake the task of data acquisition. The US landlast and sentinel series satellites are typical representatives of multispectral research. Early studies based on satellite data modeled drought environments (Theseira et al., 2002) and constructed drought early warning systems, that is, drought has been the focus of research. There have also been studies on crop leaf area monitoring (Chrysafis et al., 2020) and image classification and identification (Venkatesh and Kumar Raja, 2003; Gibson et al., 2004; Li et al., 2020b) through satellite data. Nowadays, the main research of satellites is for surface biomass (Khan et al., 2020) and monitoring of soil elements over a large area (Sullivan et al., 2004; Periasamy and Shanmugam, 2016; Ramos et al., 2020; Luo et al., 2021). In the future, as satellite technology continues to develop, the application of multispectral aspects will gradually advance, and the ability to monitor large areas as well as the types of objects to be monitored will be greatly improved.

Deep learning and machine algorithms are also extensively covered in the literature of search. The current strong emergence of UAV imaging systems has developed as a means of application in agriculture that can yield benefits. The application of machine learning algorithms (Eskandari et al., 2020; Maimaitijiang et al., 2020; Mazzia et al., 2020; Osco et al., 2020a) has improved the ability of UAV applications and likewise the ability of satellite data for agricultural applications (Datta et al., 2021). Through the study it was shown that 62% studies used regression models and 38% used classification models. With the technology development, machine learning has also gradually derived multiple medium algorithms, including artificial convolutional neural networks (Osco et al., 2020b), support vector machines (Fortin et al., 2014), etc. The early algorithms were simple development of machine vision such as detection of two-dimensional planes (Aleixos et al., 2002), for example, fruit integrity detection (Ariana et al., 2006). The integration of algorithms and equipment has achieved three-dimensional breakthroughs, for example, extraction of tree canopy volume (Li et al., 2020a; Minařík et al., 2020), etc. Currently in the field of remote sensing support vector machines and integrated classifiers are the focus of current development, while deep learning in the field of agricultural multispectral research mainly analyzes images, including image fusion, segmentation, recognition, target detection, obia, supervised training, etc. With the progress of hardware technology, the consequent acquisition of numerous data will also be beneficial to the development of machine learning, and currently big data cloud computing and machine learning are combined together to work on the development of agriculture. Deep learning is being applied to various fields of remote sensing.

In terms of multispectral detection equipment, it lacks of cooperation of ground and UAV and other aerial equipment. In some special terrain areas, the UAVs cannot shoot clearly. In this case, ground equipment will be able to make up for this shortcoming. The cooperation of multiple machinery and equipment in the future may be the focus of development research. Especially in crop modeling, UAVs and scanning instruments can cooperate with each other to combine mathematical models, build internal and external relationships, and generate complete plant images.

At present, it is difficult to achieve the simultaneous existence of multispectral and hyperspectral data on the same machine. The use of multispectral data to simulate hyperspectral data to obtain different data types by image fusion technology will be a valuable research direction in the future. Image fusion techniques can fuse different data types, not only multispectral data. The multispectral data can be extracted from the hyperspectral data or inverse performance data by specific methods such as mathematical equations, but this method is still incomplete and the current research is still focused on traditional linear algorithms. Future research should focus on studying new models or improving the accuracy by further improving machine algorithms.

UAVs play a great role with their unique advantages as the current main application platform of multispectral in agriculture. The most prominent problem with UAVs at present is the endurance problem. In the future, high endurance UAVs should be launched, while stability performance, detection accuracy, and load capacity are also necessary performance enhancements. In addition, economic and efficient multispectral equipment is needed as well. The simplicity of operation can reduce the user’s learning costs. At the same time, sensor fusion technology can also improve its application level, eliminate the possible contradictory data between sensors, reduce uncertainty, and improve the speed and correctness of the system.

Real-time image processing technology can eliminate the time gap between data collection and data analysis, and provide the basis for real-time control of operating equipment. Along with the rapid development of deep learning technology, the research on agricultural information analysis based on artificial intelligence has opened the era of intelligent research on unmanned farms. However, in the actual application operation, the uneven network coverage in the field limits the real-time transmission of images in the cloud, and the current computing performance of embedded chips also limits the real-time processing of images at the edge. Therefore, this technology is still in the early stage of research. In the future, with the popularization of 5G signal, the improvement of embedded chip computing power and the breakthrough of lightweight network model, the feasibility of real-time image processing will be gradually improved, and the foundation for the realization of unmanned and intelligent farms will be laid.

## Conclusion

The bibliometrics and scientometric approach were applied to analyze the publications on multispectral research in agriculture from 2002 to 2021. It can be seen that the number of publications in agricultural multispectral research has a rapid growth trend. The growth rate is obviously significant in recent years, maintaining a high growth rate of the literature, which is closely related to the development of precision agriculture. The study shows that the United States, China, and Spain are the countries with the largest research share, with the Chinese Academy of Sciences from China being the institution with the most publications. The most influential journal is Remote Sensing with Mutanga, Dube.timothy, and Chenghai Yang as core authors.

Multispectral technology has undergone nearly 50 years of development from the launch of ground-accessible land satellites in the United States in the 1970s to the present. It is gradually developing from the initial exploration stage to the present mature model with commercialized equipment and system software, and spreading to multiple industries. Based on the analysis, the development of agricultural multispectral technology from 2002 to 2021 is accompanied by advanced sensors and sophisticated algorithms and numerical models in agriculture. Especially along with the development of UAV technology, the application of multispectral technology in agriculture has become more and more extensive, from organic matter monitoring such as crops, to inorganic matter monitoring such as soil, moisture, nitrogen elements. The future development prospect is also more extensive. The advancement of technology has jointly promoted the application of multi-spectrum in agriculture, and also promoted the development of precision agriculture.

## Data availability statement

The raw data supporting the conclusions of this article will be made available by the authors, without undue reservation.

## Author contributions

YZ and YL: conceptualization, project administration, and funding acquisition. DZ, XHu, and YZ: methodology. DZ: software, data curation, and visualization. DZ and YZ: validation and investigation. DZ and HL: formal analysis. YZ: resources. DZ, HL, and YZ: writing—original draft preparation. JD, RJ, XHe, and MT: writing—review and editing. YL: supervision. All authors contributed to the article and approved the submitted version.

## Funding

This research was funded by Key Field Research and Development Plan of Guangdong Province, China, grant number 2019B020221001, Science and Technology Plan Project of Guangdong Province, China, grant number 2018A050506073, Guangdong Modern Agricultural Industry Generic Key Technology Research and Development Innovation Team Project, grant number 2021KJ133, and the 111Project, grant number D18019.

## Conflict of interest

The authors declare that the research was conducted in the absence of any commercial or financial relationships that could be construed as a potential conflict of interest.

## Publisher’s note

All claims expressed in this article are solely those of the authors and do not necessarily represent those of their affiliated organizations, or those of the publisher, the editors and the reviewers. Any product that may be evaluated in this article, or claim that may be made by its manufacturer, is not guaranteed or endorsed by the publisher.

## Footnotes

^1^ https://sourceforge.net/projects/citespace/

^2^ https://www.vosviewer.com/
